# Plasmid-based high-resolution melting analysis for accurate detection of *rpoB* mutations in *Mycobacterium tuberculosis* isolates from Moroccan patients

**DOI:** 10.1186/s12879-017-2666-4

**Published:** 2017-08-07

**Authors:** El Mehdi Bentaleb, My Driss El Messaoudi, Mohammed Abid, Malika Messaoudi, Ali K. Yetisen, Hassan Sefrioui, Saaïd Amzazi, Hassan Ait Benhassou

**Affiliations:** 1 Medical Biotechnology Center, Moroccan Foundation for Advanced Science, Innovation and Research (MAScIR), Rabat Design Center, Avenue Mohamed El Jazouli - Madinat Al Irfane, 10100 Rabat, Morocco; 2 Laboratory of Tuberculosis, Pasteur Institute of Morocco, Casablanca, Morocco; 3 Laboratory of Mycobacteria Genetics, Pasteur Institute of Morocco, Tangier, Morocco; 4 Harvard−MIT Division of Health Sciences and Technology, Harvard University and Massachusetts Institute of Technology, 65 Landsdowne Street, Cambridge, MA 02139 USA; 5 Laboratory of Biochemistry and Immunology, Faculty of Sciences, Mohammed V University, Rabat, Morocco

**Keywords:** Tuberculosis, Drug resistance, qPCR-HRM, Plasmid-based controls, *Mycobacterium tuberculosis*

## Abstract

**Background:**

Rapid diagnosis of drug resistance in tuberculosis (TB) is pivotal for the timely initiation of effective antibiotic treatment to prevent the spread of drug-resistant strains. The development of low-cost, rapid and robust methods for drug-resistant TB detection is highly desirable for resource-limited settings.

**Methods:**

We report the use of an in *house* plasmid-based quantitative polymerase chain reaction-high-resolution melting (qPCR-HRM) analysis for the detection of mutations related to rifampicin-resistant *Mycobacterium tuberculosis* (MTB) in clinical isolates from Moroccan patients. Five recombinant plasmids containing predominant mutations (S531L, S531W, H526Y and D516V) and the wild-type sequence of the Rifampicin Resistance-Determining Region (RRDR) have been used as controls to screen 45 rifampicin-resistant and 22 rifampicin-susceptible MTB isolates.

**Results:**

The sensitivity and the specificity of the qPCR-HRM analysis were 88.8% and 100% respectively as compared to rifampicin Drug Susceptibility Testing (DST). The results of qPCR-HRM and DNA sequencing had a concordance of 100%.

**Conclusion:**

Our qPCR-HRM assay is a sensitive, accurate and cost-effective assay for the high-throughput screening of mutation-based drug resistance in TB reference laboratories.

## Background

Tuberculosis (TB) is a major public health problem with 10.4 million new cases and 1.8 million deaths each year worldwide [1]. In developing countries, such as Morocco, the increasing incidence of drug-resistant *Mycobacterium tuberculosis* strains is one of the major factors sustaining the current TB epidemic. In 2015, Morocco registered 31,403 new TB cases, in which 1% were rifampicin resistant (RR) and multidrug-resistant tuberculosis (MDR-TB) cases [1].

Rifampicin (RIF) is a main first-line anti-TB drug and forms the backbone of short-course TB treatment in combination with isoniazid (INH). Resistance to rifampicin is caused by spontaneous mutations, which occur in the *rpoB* gene, the RNA polymerase beta-subunit encoding locus. Genetic studies have demonstrated that more than 95% of the resistance to RIF was associated with mutations in a defined 81 bp hot-spot region (codons 507–533) of the *rpoB* gene, termed rifampicin resistance-determining region (RRDR) [2–4].

Traditional phenotypic drug susceptibility tests pose delays in the detection of resistance due to the slow growth rate (days to weeks) in culturing *M. tuberculosis* specimens. Thus, rapid diagnosis of drug resistance to TB is crucial for the timely initiation of effective antibiotic treatment to prevent the spread of drug-resistant strains. Therefore, molecular-based techniques such as DNA sequencing, real-time PCR, DNA microarrays and line probe assays, have been applied to detect mutations related to TB drug resistance within 1–2 days using clinical samples or culture isolates [5–8].

The World Health Organization (WHO) has endorsed molecular line probe assays (LPA) such as INNO-LiPA® RIF TB, GenoType®MTBDR/MTBDR*plus* and GenoType® MTBDR*sl* as well as the fully automated Xpert® MTB/RIF assay and recently the Xpert® MTB/RIF Ultra for the rapid determination of genetic mutations associated with resistance to rifampicin, isoniazid and second-line anti-tuberculosis drugs [9–11]. Although these assays offer rapid analysis and high sensitivity and specificity, they have the disadvantage of being unable to cover a large panel of mutations into the *rpoB* sequence and are high cost to implement in resource-limited countries.

Recently, high-resolution melting analysis (HRMA) has been investigated for the detection of mutations such as single-nucleotide polymorphisms (SNPs) conferring drug resistance*.* This method relies on the identification of differences in PCR melting curves involving the use of dsDNA-binding dyes [12]. Several studies have evaluated HRM for the detection of resistance in cultured isolates of *M. tuberculosis* with high sensitivity and specificity as compared to conventional drug susceptibility testing in clinical practice [13]. HRM is a simple, cost-efficient and closed-tube system that requires only unlabeled primers and a dsDNA binding dye for analyzing the genotype without the need for specific probes.

The aim of the present study was to develop a plasmid-based quantitative polymerase chain reaction-HRM (qPCR-HRM) assay for accurate and cost-effective identification of RIF-resistant *M. tuberculosis* strains and to determine the main mutations conferring resistance to RIF in clinical isolates from Morocco.

## Methods

### Bacterial isolates and drug susceptibility testing

The ethics charter of the Pasteur Institute of Morocco (Casablanca, Morocco) approved the consent procedure and the protocols (IRB reference number: IPM2013-P3). The framework of the charter ensures the protection of patient’s clinical information and confidential preservation of the results. Sixty seven clinical *M. tuberculosis* isolates from TB-confirmed patients were provided by the tuberculosis reference laboratory of the Pasteur Institute of Morocco (Casablanca, Morocco). Drug susceptibility testing (DST) of the selected samples was previously established using the proportion method in Lowenstein-Jensen (L-J) medium [14]. Two groups of strains were used in this study: 45 rifampicin resistant (RIF-R) and 22 rifampicin susceptible (RIF-S) isolates.

### DNA extraction

Mycobacterial genomic DNA was extracted and purified using QIAamp DNA Mini Kit (Qiagen, USA) in accordance with the protocol provided by the manufacturer. DNA yield and purity was checked with a UV-Vis spectrophotometer (NanoDrop 2000, Thermo Scientific, Waltham, MA, USA). Purified genomic DNA was stored at −20 °C until analysis.

### Construction of reference plasmids

To evaluate the specificity of HRM discrimination among different types of mutations, five plasmid-controls were designed and constructed. These plasmids contained four of the most predominant mutations (S531L, S531W, H526Y, or D516V) and a fifth wild-type (WT) plasmid with no mutation in the 81 bp hot-spot region. For each plasmid, 377 bp segment of *rpoB* gene containing the RRDR region was synthetically generated and then cloned into a 2507 bp pEX-K vector (Eurofins MWG Operon, Germany). All the vectors were then used to transform highly competent *E. coli* (New England Biolabs, MA, USA). Transformed *E. coli* cells carrying recombinant plasmid DNA were grown overnight in Luria Bertani (LB) broth (10 mL, Sigma Aldrich, USA) containing kanamycin (50 μg mL^−1^, Invitrogen, USA) at 37 °C at 300 rpm constant shaking. The bacterial cultures were harvested by centrifugation at 6000×g for 15 min at 4 °C. The supernatant was removed and the cell pellets consisting of the recombinant plasmid were isolated and purified with Wizard® Plus SV Minipreps DNA Purification System (Promega, USA) according to the manufacturer’s instructions. The purified plasmid DNA samples were quantified and then stored at −20 °C.

### Real-time PCR and HRM analysis

To perform qPCR-HRM, primers were manually designed to specifically amplify a 120 bp amplicon spanning the RRDR (*RRDR*-Fwd, 5′-CCGCGATCAAGGAGTTCTTC-3′; *RRDR*-Rev., 5′-GTGACAGACCGCCGGG-3′). Basic Local Alignment Search Tool (BLAST) analysis (National Center for Biotechnology Information, NCBI) was performed on the primer pair to evaluate their specificity.

The reaction mixture was made up using 2X MeltDoctor HRM Master Mix (Applied Biosystems, USA) (10 μL), each primer (1.5 μL, 3 μM), genomic or plasmid DNA (5 μL) and diethyl pyrocarbonate (DEPC)-treated water (1 μL) to obtain a 20 μl final reaction volume. The staining method used in the qPCR-HRM assays was based on the SYTO9® intercalating dye. SYTO9® is a third generation dye, which is more robust to generate shaped melting curves as compared to SYBR Green I. The latter dye is known to cause PCR inhibition when used at high concentrations, leading to redistribution problems during melting stage. SYTO9® can be used at higher concentrations due to low reaction toxicity, drastically increasing dsDNA saturation and avoiding redistribution effects during DNA dissociation.

Quantitative PCR and HRM assays were performed in 96-fast well plates using an integrated QuantStudio 6 Flex System (Applied Biosystems, USA). QuantStudio™ 6 heating block has the capability to control the temperature by less than 0.1 °C increments, which offers a high melt precision.PCR cycling run under the following conditions: one cycle of 95 °C for 10 min, 40 cycles of 95 °C for 15 s and 60 °C for 1 min. HRM curve acquisition was performed during a post-PCR stage (dissociation stage). Previously amplified samples were heated to 95 °C for 10 s, cooled to 60 °C for 1 min, and then heated from 60 °C to 95 °C rising by 0.025 °C s^−1^. Analysis of the obtained curves was performed using the High Resolution Melt Module for QuantStudio™ 6 and 7 Flex Real-Time PCR System Software that allows both acquisition and analyses of PCR plots and HRM curves on the same interface. All samples were tested in duplicates including negative and positive controls and different genotypes were assigned in accordance to the HRM curves of plasmid controls.

### PCR and DNA sequencing

The primers rpoB-F 5′-GGGAGCGGATGACCACCCA-3′ and rpoB-R 5′ GCGGTACGGCGTTTCGATGAAC-3′ were previously described [15] to amplify a 350-bp segment of *rpoB* containing the RRDR region. PCR reactions were performed with a Veriti Thermal Cycler (Applied Biosystems, USA) using PCR Supermix (Invitrogen, USA) under the following cycling conditions: (i) an initial step at 95 °C for 5 min, (ii) 35 cycles at 95 °C for 45 s, 68 °C for 45 s and 72 °C for 45 s, and (iii) a 10-min step at 72 °C.

The sequencing was carried out by utilizing the BigDye Terminator v3.1 Cycle Sequencing Kit (Thermo Fisher Scientific, USA) and an Applied Biosystems 3730 DNA Analyzer from Secugen (Madrid, Spain). Constructed plasmid controls and *H37Ra* PCR products were used as an internal control to validate the results. The mutations were detected by mapping against H37Rv strain as a reference sequence obtained from GenBank (NCBI, Bethesda, MD, USA) (Accession on NC_000962). Sequence alignment was carried out by using BioEdit 7.2.5 software (Ibis Biosciences, Carlsbad, CA, USA).

### Statistical analysis

Sensitivity, specificity as well as concordance of the qPCR-HRM assay were calculated in comparison with the standard DST and DNA sequencing, respectively. Sensitivity was defined as [Number of drug-resistant isolates with mutations]/[Number of drug-resistant isolates with mutations + number of drug-resistant isolates without mutation]; and specificity as [Number of drug-susceptible isolates without mutations]/[number of drug susceptible isolates with mutations + number of drug-susceptible isolates without mutations] [16]. Concordance analysis was performed to assess the near-equivalence of the qPCR-HRM and DNA sequencing used for assessing presence/absence of mutations [17]. Concordance between the two techniques results was calculated as [Number of drug-resistant isolates with mutations + number of drug-susceptible isolates without mutations]/[total number of samples]. Sensitivity and specificity calculations were estimated at a 95% confidence interval [95% CI]. Statistical analysis was performed using SPSS 17.0 software (SPSS Inc., USA).

## Results

**Fig. 1:**
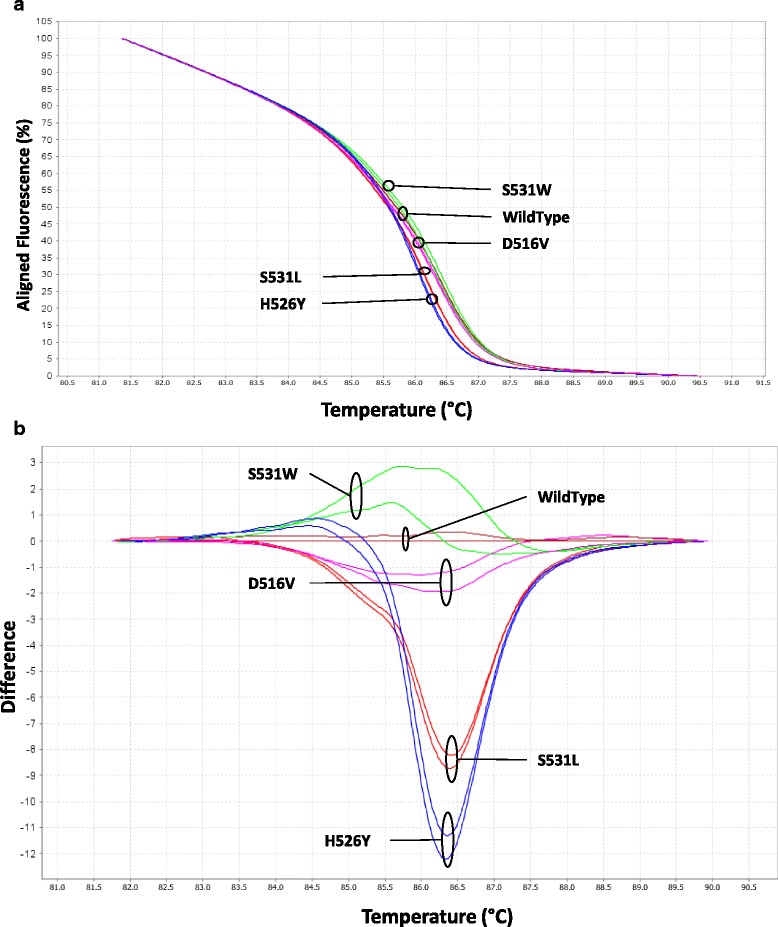
High resolution melting analysis of five plasmid-based controls. Representative aligned (**a**) and difference (**b**) curves derived from the normalized data using pEX-K WildType and *M. tuberculosis H37ra* reference strain as the baseline and four pEX-K plasmids harboring D516V, H526Y, S531W and S531L mutations

Sixty seven *M. tuberculosis* strains from TB-confirmed patients were enrolled in this study. Among them, 45 cases were phenotypically RIF-resistant and 22 cases were RIF-susceptible. In comparison to phenotypic data, *rpoB* genotyping showed that all strains harboring mutations in the RRDR were resistant to rifampicin. The *rpoB* mutations detected by HRM and confirmed by DNA sequencing were summarized in the Table 1. Amplification products from *M. tuberculosis H37ra* genomic DNA and *M. tuberculosis* RIF-susceptible isolates had the same melting profile when compared to pEX-K WT melt curve. Additionally, HRM profiles of the recombinant plasmids carrying mutations (pEX-K D516V, pEX-K H526Y, pEX-K S531L and pEX-K S531W) were accurately discriminated (Fig. 1) and labeled by the HRM software as controls.

**Table 1:** Mutations detected by qPCR-HRM and confirmed by DNA sequencing

Rifampicin phenotypic profile	Amino Acid Change	Nucleotide Change	Number of mutations (Percentage)	qPCR-HRM assay	DNA Sequencing
Resistant (45)	S531L	TCG>TTG	32 (71.1%)	Mutant	Mutant
	S531W	TCG>TGG	2 (4.4%)	Mutant	Mutant
	H526Y	CAC>TAC	2 (4.4%)	Mutant	Mutant
	H526L	CAC>CTC	1 (2.2%)	Mutant	Mutant
	H526C	CAC>TGC	3 (6.7%)	Mutant	Mutant
	No mutation	-	5 (11.1%)	Wild	Wild
Susceptible (22)	No mutation	-	--	Wild	Wild

**Fig. 2:**
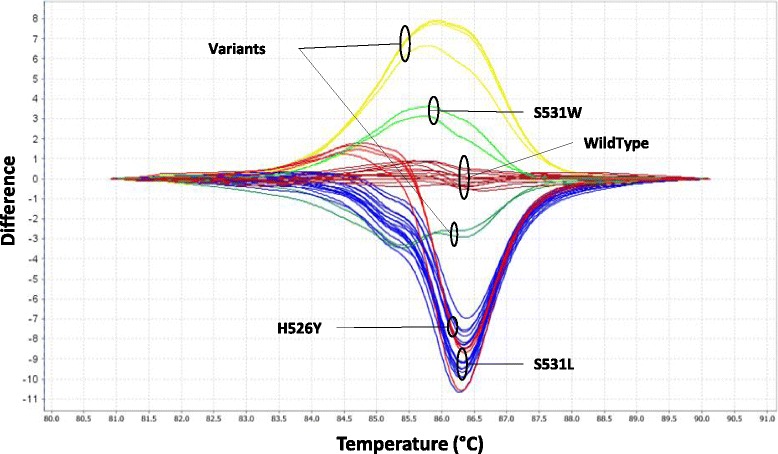
Plasmid-based high resolution melting analysis of *M. tuberculosis* isolates. Differential plots of the 120 bp segment of the *rpoB* gene from *Mycobacterium tuberculosis* isolates. The five plasmid controls melting profiles were set up as reference control groups. The baseline represents the pEX-K WT, *M. tuberculosis H37ra* and RIF-S isolates, as well as five RIF-R isolates. Melting profile genotypes were indicated by matching to previously set control groups. The other melt curves including mutations were clearly distinguishable and control groups were defined as variants

All *M. tuberculosis* strains carrying H526Y, S531L and S531W were correctly defined in control groups, with the exception of D516V group, as no D516V mutation was found in all analyzed *M. tuberculosis* rifampicin-resistant isolates. The five plasmid-based controls had a specificity of 100%, and the plasmid control pEX-K WT differentiated all WT genotypes from those including single or double mutations. Furthermore, all strains presenting the S531L, S531W and H526Y changes were identified and sorted according to their corresponding plasmid controls and no strains with other mutations (variants) were included in control groups. Additionally, two mutations have been classified in variant groups 1 and 2 (Fig. 2) and were then identified by DNA sequencing as single mutation H526L (CAC/CTC) and double mutation H526C (CAC/TGC), respectively.

Among 45 genotypically typed RIF-resistant isolates, the mutation S531L (TCG/TTG) was the most encountered (71.1%, 32/45), followed by H526C (CAC/TGC) (6.7%, 3/45), S531W (TCG/TGG) and H526Y (CAC/TAC) (equally 4.4%, 2/45) and H526L (CAC/CTC) (2.2%, 1/45) (Table 1). In addition, no deletions or insertions were detected. However, 5/45 RIF resistant *M. tuberculosis* isolates contained no mutation in the *rpoB* amplified region and were classified as phenotypically RIF-resistant isolates.

HRMA identified 40 of 45 isolates that were resistant and 22/22 (100%) that were susceptible on DST by L-J proportion method with a sensitivity and specificity of 88.8% (95% CI: 75.95–96.29) and 100% (95% CI: 84.56–100.00), respectively. The concordance between HRMA and DNA sequencing was 100%, where the 40 genotypically RIF-resistant isolates were correctly categorized as single or double mutations in the *RRDR* region while all the 22 RIF-susceptible isolates were classified as WT.

## Discussion

Investigating the prevalence of geography-specific mutations is important for the development of *in-house* qPCR-HRM assays targeting relevant mutations in each specific setting. In this study, we have developed and evaluated a qPCR-HRM assay for accurate detection of mutations mostly associated with resistance to rifampicin. All mutations in the resistant isolates had specific melt curve patterns and were further characterized by DNA sequencing. In accordance with the previous reports from Lebanon, Iran/Iraq, Kuwait, United Arab Emirates, China, Belarus, Honduras, Romania, and Uganda [18–21], the most frequently occurring mutations detected in our study were at codon 531 (75.5%), followed by codon 526 (13.3%). In comparison to the reports from Morocco [22–26], mutations at codons 531 had relative frequencies varying from 49.8% to 83.6%. The high frequency of codon 531 mutant isolates could be due to the spread of an endemic clone or a biased sampling due to a relatively small number of isolates. Notably, a double mutation at codon 526 (H526C) was observed in Moroccan isolates with a relatively high frequency (6.7%, 3/45). This mutation was previously reported in Moroccan [27], Turkish [28], Chinese & German [29], and Korean [30] isolates with low frequencies.

Our study confirmed the accuracy of plasmid-based controls for *rpoB* mutation detection by qPCR-HRM as previously reported by others [31, 32]. Including the plasmid controls pEX-K S531W and pEX-K D516V in the qPCR-HRM assay has demonstrated the high sensitivity of the assay in discriminating resistant isolates carrying SNP class 3 and 4, which were the most challenging mutations (C/G and A/T, respectively). In these SNP classes, bases switch only their specific strands, resulting in low melting temperatures differences (∆Tm) that are difficult to identify, which can lead to false negative results [33]. In addition, the use of plasmid-based controls was proven to be a valuable alternative to genomic DNA as it reduced the cost of the assay as compared to the high-cost, labor-intensive and time-consuming preparation of reference strains DNA that requires biosafety level (BSL-3) facilities [32, 34]. Moreover, the plasmid-based controls allow not only the detection of the drug-resistant TB presence, but also the identification of the nature of *rpoB* mutation. Thus, this permits the establishment of an accurate diagnosis of RIF resistance in case of mutation occurrence associated with different levels of rifampicin resistance [35, 36]. It could be also useful for an absolute quantitative detection when pathogen copy number is necessary for disease and treatment control.

According to our results, there was phenotypic and genotypic concordance in 40 out of 45 resistant isolates. All genotypes determined by qPCR-HRM were confirmed by DNA sequencing with a concordance of 100%. The sensitivity (88.8%) and specificity (100%) of the qPCR-HRM assay in the present study are consistent with previously published studies using HRM in clinical isolates [31, 37–41]. Only five phenotypically resistant isolates were genotypically susceptible (WT), which has affected the sensitivity of the assay. For these strains, other mutations might have occurred outside the *rpoB* region examined in the present work. Although resistance to rifampicin is mostly mediated by mutations located in the 81-bp RRDR of *rpoB* gene, other mutations can be occasionally associated with resistance to rifampicin. Several studies in various geographic locations have reported mutations outside the RRDR hot spot: (GTC/TTC) at codon 176 [42] and (GCG/GTG) at codon 381 [43]. Mutation at codon 146 (V to F) was found frequently in *M. tuberculosis* strains recovered from patients of Middle Eastern origin [44]. However, it is speculated that additional mechanisms such as low cell wall permeability, the presence of membrane proteins acting as drug efflux pumps or the production of drug-modifying and inactivating enzymes can contribute to rifampicin resistance in *Mycobacteria* [45, 46].

Our study results have confirmed the performance of HRMA as a method for the rapid and accurate detection of mutations associated to MDR-TB as previously reported by studies that have successfully discriminated resistant strains from clinical isolates [31, 37–40]. Furthermore, in a recent study, Sharma et al. have evaluated HRM curve analysis as a rapid approach for the diagnosis and screening of rifampicin resistance in tuberculous meningitis directly from cerebrospinal fluid (CSF) samples [47]. More recently, Anthwal et al. have reported the use of a plasmid-based HRM assay for direct detection of MDR-TB in sputum, especially for direct smear negative cases [32]. These studies suggest that HRMA can be a robust, accurate, sensitive and cost-effective diagnostic technology for the detection of MDR-TB on both clinical isolates and patient samples.

## Conclusion

This is the first study of developing and evaluating a plasmid-based qPCR-HRM assay for rifampicin resistance screening in cultured isolates from Moroccan TB patients. The results indicate that this assay has the potential to be a valuable high-throughput, accurate and cost-effective technology in TB reference laboratories. However, further evaluation of our qPCR-HRM assay directly on sputum samples would be crucial to shorten the delay in drug resistance diagnosis. Moreover, screening the *rpoB* gene in a larger population can reveal new region-specific mutations that may occur out of RRDR. Thus, covering other regions in the *rpoB* gene would enable the detection of unidentified mutations by building a library of plasmid controls harboring frequently occurring mutations.

## Abbreviations

DEPC: Diethyl Pyrocarbonate
dsDNA: Double Stranded deoxyribonucleic acid
DST: Drug Susceptibility Testing
INH: Isoniazid
LB: Luria Bertani
L-J: Lowenstein-Jensen
MDR-TB: Multidrug-resistant tuberculosis
MTB: *Mycobacterium tuberculosis*
qPCR-HRM: quantitative Polymerase Chain Reaction-High-Resolution Melting
RIF: Rifampicin
*rpoB*: RNA polymerase beta-subunit
RR: Rifampicin Resistant
RRDR: Rifampicin Resistance-Determining Region
SNP: Single Nucleotide Polymorphism
TB: Tuberculosis
